# Quantum algorithms for topological and geometric analysis of data

**DOI:** 10.1038/ncomms10138

**Published:** 2016-01-25

**Authors:** Seth Lloyd, Silvano Garnerone, Paolo Zanardi

**Affiliations:** 1 Department of Mechanical Engineering, Research Lab for Electronics, Massachusetts Institute of Technology, MIT 3-160, Cambridge, Massachusetts 02139, USA; 2 Institute for Quantum Computing, University of Waterloo, Waterloo, Ontario, Canada N2L 3G1; 3 Department of Physics and Astronomy, Center for Quantum Information Science & Technology, University of Southern California, Los Angeles, California 90089-0484, USA

## Abstract

Extracting useful information from large data sets can be a daunting task. Topological methods for analysing data sets provide a powerful technique for extracting such information. Persistent homology is a sophisticated tool for identifying topological features and for determining how such features persist as the data is viewed at different scales. Here we present quantum machine learning algorithms for calculating Betti numbers—the numbers of connected components, holes and voids—in persistent homology, and for finding eigenvectors and eigenvalues of the combinatorial Laplacian. The algorithms provide an exponential speed-up over the best currently known classical algorithms for topological data analysis.

## Abstract (web summary)

Persistent homology allows identification of topological features in data sets, allowing the efficient extraction of useful information. Here, the authors propose a quantum machine learning algorithm that provides an exponential speed up over known algorithms for topological data analysis.

## Graphical abstract

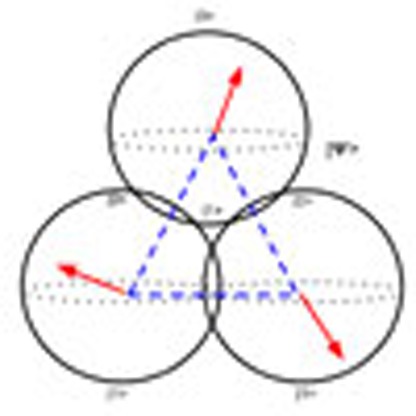

Human society is currently generating on the order of Avogadro's number (6 × 10^23^) of bits of data a year. Extracting useful information from even a small subset of such a huge data set is difficult. A wide variety of big data processing techniques have been developed to extract from large data sets the hidden information in which one is actually interested. Topological techniques for analysing big data represent a sophisticated and powerful tool^1^,^2^,^3^,^4^,^5^,^6^,^7^,^8^,^9^,^10^,^11^,^12^,^13^,^14^,^15^,^16^,^17^,^18^,^19^,^20^,^21^,^22^,^23^,^24^. By its very nature, topology reveals features of the data that robust to how the data were sampled, how it was represented and how it was corrupted by noise. Persistent homology is a particularly useful topological technique that analyses the data to extract topological features such as the number of connected components, holes, voids and so on (Betti numbers) of the underlying structure from which the data was generated. The length scale of analysis is then varied to see whether those topological features persist at different scales. A topological feature that persists over many length scales can be identified with a ‘true' feature of the underlying structure.

Topological methods for analysis face challenges: a data consisting of *n* data points possesses 2^*n*^ possible subsets that could contribute to the topology. Performing methods of algebraic topology on simplicial complexes eventually requires matrix multiplication or diagonalization of matrices of dimension 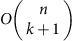 to extract topological features at dimension *k*. For small *k*, such operations require time polynomial in *n*; however, to extract high-dimensional features, matrix multiplication and diagonalization lead to problem solution scalings that grow exponentially in the size of the complex. A variety of mathematical methods have been developed to cope with the resulting combinatorial explosion, notably mapping the complex to a smaller complex with the same homology, and then performing the matrix operations on the reduced complex^1^,^2^,^3^,^4^,^5^,^6^,^7^,^8^,^9^,^10^,^11^,^12^,^13^,^14^,^15^,^16^,^17^,^18^,^19^,^20^,^21^,^22^,^23^,^24^. Even in such cases, the initial reduction must identify all simplices in the original complex, and so can scale no better than linearly in the number of simplices. Consequently, even with only a few hundred data points, creating the persistent homology for Betti numbers at all orders of *k* is a difficult task. In particular, the most efficient classical algorithms for estimating Betti numbers at order *k* (the number of *k*-dimensional gaps, holes and so on), have computational complexity either exponential in *k* or exponential in *n* (refs 7, 8, 9, 10, 11, 12), so that estimating Betti numbers to all orders scales exponentially in *n*, and algorithms for diagonalizing the combinatorial Laplacian (that reveal not only the Betti numbers but additional geometric structure) at order *k* have computational complexity as 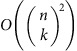, where *n* is the number of vertices in the (possibly reduced) complex. That is, the best classical algorithms for estimating Betti numbers to all orders^9^,^10^,^11^,^12^ and for diagonalizing the full combinatorial Laplacian grow exponentially in the number of vertices in the complex.

This paper investigates quantum algorithms for performing topological analysis of large data sets. We show that a quantum computer can find the eigenvectors and eigenvalues of the combinatorial Laplacian and estimate Betti numbers to all orders and to accuracy *δ* in time *O*(*n*^5^/*δ*), thereby reducing a classical problem for which the best existing solutions have exponential computational complexity, to a polynomial-time quantum problem. Betti numbers can also be estimated by using a reduced, or ‘witness' complex, that contains fewer points than the original complex^1^,^2^,^3^,^4^,^5^,^6^,^7^,^8^,^9^,^10^,^11^,^12^. Applied to such witness complexes, our method again yields a reduction in estimation time from 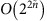 to 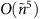, where 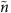 is the number of points in the reduced complex.

Recently, quantum mechanical techniques have been proposed for machine learning and data analysis^25^,^26^,^27^,^28^,^29^,^30^,^31^,^32^,^33^,^34^. In particular, some quantum machine learning algorithms^31^,^32^,^33^ provide exponential speed-ups over the best existing classical algorithms for supervised and unsupervised learning. Such ‘big quantum data' algorithms use a quantum random access memory (qRAM)^35^,^36^,^37^ to map an *N*-bit classical data set onto the quantum amplitudes of a (log_2_ *N*)-qubit quantum state, an exponential compression over the classical representation. The resulting state is then manipulated using quantum information processing in time poly(log_2_ *N*) to reveal underlying features of the data set. That is, quantum computers that can perform ‘quantum sampling' of data can perform certain machine learning tasks exponentially faster than classical computers performing classical sampling of data. A discussion of computational complexity in quantum machine learning can be found in ref. 34. Constructing a large-scale qRAM to access *N∼*10^9^−10^12^ pieces of data is a difficult task. By contrast, the topological and geometrical algorithms presented here do not require a large-scale qRAM: a qRAM with *O*(*n*^2^) bits suffices to store all pairwise distance information between the points of our data set. The algorithms presented here obtain their exponential speed-up over the best existing classical algorithms not by having quantum access to a large data set, but instead, by mapping a combinatorially large simplicial complex with *O*(2^*n*^) simplices to a quantum state with *n* qubits, and by using quantum information processing techniques such as matrix inversion and diagonalization to perform topological and geometrical analysis exponentially faster than classical algorithms. Essentially, our quantum algorithms operate by finding the eigenvectors and eigenvalues of the combinatorial Laplacian. But diagonalizing a 2^*n*^ by 2^*n*^ sparse matrix using a quantum computer takes time *O*(*n*^2^), compared with time *O*(2^2*n*^) on a classical computer^38^,^39^,^40^.

The algorithms given here are related to quantum matrix inversion algorithms^41^. The original matrix inversion algorithm^41^ yielded as solution a quantum state, and left open the question of how to extract useful information from that state. The topological and geometric algorithms presented here answer that question: the algorithms yield as output not quantum states but rather topological invariants—Betti numbers—and do so in time exponentially faster than the best existing classical algorithms. The best classical algorithms for calculating the *k*th Betti number takes time *O*(*n*^*k*^), and estimating Betti numbers to all orders to accuracty *δ* takes time at least *O*(2^*n*^ log(1/*δ*)) (refs 7, 8, 9, 10, 11, 12). Exact calculation of Betti numbers for some types of topological sets (algebraic varieties) is PSPACE hard^42^. By contrast, our algorithm provides approximate values of Betti numbers to all orders and to accuracy *δ* in time *O*(*n*^5^/*δ*): although no polynomial classical algorithm for such approximate evaluation of topological invariants is known, the computational complexity of such approximation remains an open problem. We do not expect our quantum algorithms to solve a PSPACE-hard problem in polynomial time. We summarize the comparison between the amount of resources required by the classical and quantum algorithms in Table 1.

## Results

### The quantum pipeline

The quantum algorithm operates by mapping vectors, simplices, simplicial complexes and collections of simplicial complexes to quantum mechanical states, and reveals topology by performing linear operations on those states. The 2^*n*^ possible simplices of the simplicial complex are mapped onto an *n*-qubit quantum state. This state is then analysed using conventional quantum computational techniques of eigenvector and eigenvalue analysis, matrix inversion and so on. The quantum analysis reveals topological features of the data, and shows how those features arise and persist when the scale of analysis is varied. The resulting quantum algorithms provide an exponential speed-up over the best existing classical algorithms for topological data analysis.

In addition to revealing topological features such as Betti numbers, our algorithm uses the relationship between algebraic topology and Hodge theory^9^,^10^,^11^,^12^,^14^,^15^,^16^,^17^,^18^,^19^,^20^,^21^,^22^,^23^,^24^ to reveal geometrical information about the data analysed at different scales. The algorithm operates by identifying the harmonic forms of the data, together with the other eigenvalues and eigenvectors of the combinatorial Laplacian—the quantities that famously allow one to ‘hear the shape of a drum'^43^. The quantum algorithm reveals these geometric features exponentially faster than the corresponding classical algorithms. In particular, our quantum algorithm for finding all Betti numbers for the persistent homology for simplicial complexes over *n* points and for diagonalizing the combinatorial Laplacian takes time *O*(*n*^5^/*δ*), where *δ* is the multiplicative accuracy to which Betti numbers and eigenvalues are determined. The best available classical algorithms to perform these tasks at all orders of *k* take time *O*(2^2*n*^ log(1/*δ*)).

The advantage of big quantum data techniques is that they provide exponential compression of the representation of the data. The challenge is to see if—and this is a big ‘if'—it is still possible to process the highly compressed quantum data to reveal the desired hidden structure that underlies the original data set. Here we show that quantum information processing acting on large data sets encoded in a quantum form can indeed reveal topological features of the data set.

Classical algorithms for persistent homology have two steps (the ‘pipeline'). First, one processes the data to allow the construction of a topological structure such as a simplicial complex that approximates the hidden structure from which the data was generated. The details of the topological structure depends on the scale at which data is grouped together. Second, one constructs topological invariants of that structure and analyses how those invariants behave as a function of the grouping scale. As above, topological invariants that persist over a wide range of scales are identified as features of the underlying hidden structure.

The quantum ‘pipeline' for persistent homology also has two steps. First, one accesses the data in quantum parallel to construct quantum states that encode the desired topological structure: if the structure is a simplicial complex, for example, one constructs quantum states that are uniform superposition of descriptions of the simplices in the complex. Second, one uses the ability of quantum computing to reveal the ranks of linear maps to construct the topological invariants of the structure. The steps of the quantum pipeline are now described in more detail.

### Constructing a simplicial complex

Classical persistent homology algorithms use the access to data and distances to construct a topological structure—typically a simplicial complex—that corresponds to the hidden structure whose topology one wishes to reveal. In the quantum algorithm, we use the ability to access data and to estimate distances in quantum parallel to construct quantum states that encode the simplicial complex. Each simplex in the complex consists of a fully connected set of vertices: a *k*-simplex *s*_*k*_ consists of *k*+1 vertices *j*_0_, *j*_1_, …, *j*_*k*_ (listed in ascending order, *j*_0_<*j*_1_< … <*j*_*k*_) together with the *k*(*k*+1)/2 edges connecting each vertex to all the other vertices in the simplex. Encode a *k*-simplex *s*_*k*_ as a string of *n* bits, for example, 0110 … 1, with *k*+1 1s at locations *j*_0_, *j*_1_, …, *j*_*k*_ designating the vertices in the simplex. Removing the 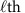 vertex and its associated edges from a *k*-simplex yields a *k*−1 simplex. The *k*+1 simplices 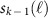 with vertices 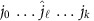 obtained by removing the 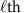 vertex 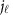 from *s*_*k*_ form the boundary of the original simplex. The number of potential simplices in a simplicial complex is equal to 2^*n*^, the number of possible subsets of the *n* points in the graph. That is, every member of the power set is a potential simplex. If *n* is large, the resulting combinatorial explosion means that identifying large simplices can be difficult.

To define a simplicial complex, fix a grouping scale *ε*, and identify *k* simplices as subsets of *k*+1 points that are all within *ε* of each other. The resulting set of simplices *S*^*ε*^ is called the Vietoris–Rips complex. The form of the simplicial complex *S*^*ε*^ depends on the scale *ε* at which its points are grouped together: persistent homology investigates how topological invariants of the simplicial complex depend on the scale *ε*. The collection of simplicial complexes {*S*^*ε*^} for different values of the grouping scale *ε* is called a filtration. Note that if a simplex belongs to the complex *S*^*ε*^, then it also belongs to *S*^*ε*′^, *ε*′>*ε*. That is, the filtration consists of a sequence of nested simplicial complexes. When *ε* is sufficiently small, only the zero-simplices (points) lie in the complex. As *ε* increases, one and two simplices (edges and triangles) enter the complex, followed by higher order simplices. As *ε* continues to increase, topological features such as holes, gaps and voids come into existence, and then are eventually filled in. For sufficiently large *ε*, all possible simplices are contained in the complex.

Now construct quantum states that correspond to the simplicial complex. Encode simplices as quantum states over *n* qubits with 1 s at the positions of the vertices. We designate the *k*-simplex *s*_*k*_ by the *n*-qubit basis vector 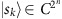. Denote the 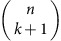 dimensional Hilbert space corresponding to all possible *k* simplices by *W*_*k*_. Let 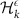 be the subspace of *W*_*k*_ spanned by 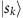 where 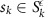, the set of *k* simplices in *S*^*ε*^. The full simplex space at scale *ε* is defined to be 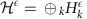. Assume that the distances between pairs of points are either given by a quantum algorithm or stored in qRAM (see Methods section). The ability to evaluate distances translates onto the ability to apply the projector 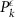 that projects onto the *k*-simplex space 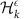
and the projector *P*^*ε*^ that projects onto the full simplex space 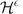.

Grover's algorithm can then be used to construct the *k*-simplex state


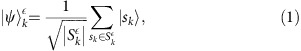


where as above 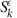 is the set of *k* simplices in the complex at scale *ε*. That is, 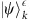 is the uniform superposition of the quantum states corresponding to *k* simplices in the complex. For each simplex *s*_*k*_, we can verify whether 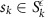 in *O*(*k*^2^) steps. That is, we can implement a membership function 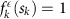 of 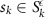 in *O*(*k*^2^) steps. The multi-solution version of Grover's algorithm then allows us to construct the *k*-simplex state of equation (1).

The construction of the *k*-simplex state via Grover's algorithm reveals the number of *k* simplices 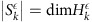 in the complex at scale *ε*, and takes time 
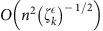
, where 
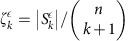
 is the fraction of possible *k* simplices that are actually *n* the complex at scale *ε*. When this fraction is too small, the quantum search procedure will fail to find the simplices. For *k*<<*n*, we have 
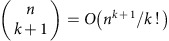
, and 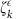 is only polynomially small in *n*. By contrast, for *k*≈*n*, ζ_*k*_^*ε*^ can be exponentially small in *n*: if only an exponentially small set of possible simplices actually lie in the complex, quantum search will fail to find them. For the purposes of performing the quantum algorithm, we fix a parameter ζ that determines the accuracy to which we wish to determine the simplex state, and run the simplex finding algorithm for a time ζ^−1/2^. At each grouping scale *ε*, the algorithm will find *k* simplices when 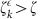, and estimate the number of *k* simplices to accuracy 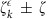. As *ε* increases, more and more simplices enter into the complex; 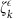 increases; and quantum search will succeed in constructing the simplex state to greater and greater accuracy. When *ε* becomes larger than the maximum distance between vectors, all simplices are in the complex.

Below, it will prove useful to have, in addition to the simplex state 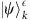 the state 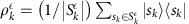, which is the uniform mixture of all *k*-simplex states in the complex at grouping scale *ε*. 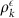 can be constructed in a straightforward fashion from the simplex state 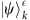 by adding an ancilla and copying the simplex label to construct the state 
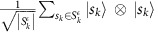
. Tracing out the ancilla then yields the desired uniform mixture over all *k* simplices.

In summary, we can represent the the simplicial complex in quantum mechanical form using exponentially fewer bits than that are required classically. Indeed, the quantum search method for constructing simplicial states works best when 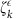 is not too small, so that a substantial fraction of simplices that could be in the complex are actually in the complex. But this regime is exactly the regime where the classical algorithms require an exponentially large amount of memory space bits merely to record which simplices are in the complex. Now we show how to act on this quantum mechanical representation of the filtration to reveal persistent homology.

### Topological analysis

Having constructed a quantum state that represents the simplicial complex *S*^*ε*^ at scale *ε*, we use quantum information processing to analyse its topological properties. In algebraic topology in general, and in persistent homology in particular, this analysis is performed by investigating the properties of linear maps on the space of simplices. As above, let 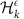 be the Hilbert space spanned by vectors corresponding to *k* simplices in the complex at level *ε*. We identify the vector space 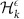 with the abelian group *C*_*k*_ (the *k*th chain group) under addition of vectors in the space. Let *j*_0_ … *j*_*k*_ be the vertices of *s*_*k*_. Define the boundary map *∂*_*k*_ on the space of *k* simplices by


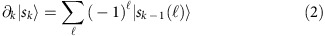


where as above 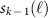 is the *k*−1 simplex on the boundary of *s*_*k*_ with vertices 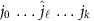 obtained by omitting the 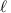
th vertex 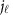 from *s*_*k*_. The boundary map maps each simplex to the oriented sum of its boundary simplices. *∂*_*k*_ is a 
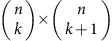
 matrix with *n*−*k* non-zero entries ±1 in each row and *k*+1 non-zero entries ±1 per column. Note that *∂*_*k*_*∂*_*k*+1_=0: the boundary of a boundary is zero. As defined, *∂*_*k*_ acts on the space of all *k* simplices. We define the boundary map restricted to operate from 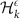 to 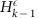 to be 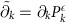, where as above 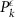 is the projector onto the space of *k* simplices in the complex at scale *ε*.

The *k*th homology group **H**_*k*_ is the quotient group, 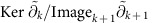, the kernel of 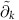 divided by the image of 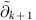 acting on 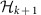
at grouping scale *ε*. The *k*th Betti number *β*_*k*_ is equal to the dimension of **H**_*k*_, which in turn is equal to the dimension of the kernel of 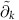 minus the dimension of the image of 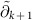.

The strategy that we use to identify persistent topological features operates by identifying the singular values and singular vectors of the boundary map. Connected components, holes, voids and so on, correspond to structures—chains of simplices—that have no boundary, but that are not themselves a boundary. That is, we are looking for the set of states that lie within the kernel of 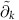, but that do not lie within the image of 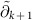. The ability to decompose arbitrary vectors in 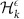 in terms of these kernels and images allows us to identify Betti numbers at different grouping scales *ε*.

The quantum phase algorithm^38^,^39^,^40^ allows one to decompose states in terms of the eigenvectors of an Hermitian matrix and to find the associated eigenvalues. Once the *k*-simplex states 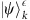 have been constructed, the quantum phase algorithm allows one to decompose those states in terms of eigenvectors and eigenvalues of the boundary map. The boundary map is not Hermitian. We embed the boundary map 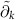 into a Hermitian matrix 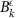 defined by


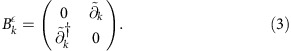



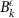
 acts on the space 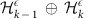. Note that 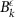 is *n*-sparse: there are either *k* or *n*−*k* entries per row. Similarly, define the full Hermitian boundary map to be


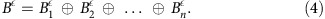


*B*^*ε*^ is also *n*-sparse. Because 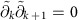, we have 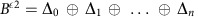, where 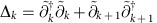 is the combinatorial Laplacian of the *k*th simplicial complex^22^,^23^,^24^. Because 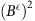 is the sum of the combinatorial Laplacians, *B*^*ε*^ is sometimes called the ‘Dirac operator', since the original Dirac operator was the square root of the Laplacian. Explicit matrix forms of the Dirac operator and the combinatorial Laplacian are given in the Methods section. Hodge theory^9^,^10^,^11^,^12^,^14^,^15^,^16^,^17^,^18^,^19^,^20^,^21^,^22^,^23^,^24^ implies that the *k*th homology group satisfies 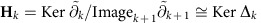. The dimension of this kernel is the *k*th Betti number.

To find the dimension of the kernel, apply the quantum phase algorithm^38^,^39^,^40^ to *B*^*ε*^ starting from the uniform mixture of simplices *ρ*^*ε*^. The quantum phase algorithm decomposes this state into the eigenvectors of the combinatorial Laplacian, and identifies the corresponding eigenvalues. The probability of yielding a particular eigenvalue is proportional to the dimension of the corresponding eigenspace. As above, classical algorithms for finding the eigenvalues and eigenvectors of the combinatorial Laplacians Δ_*k*_, and calculating the dimension of the eigenspaces takes 
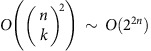
 computational steps using sparse matrix diagonalization via Gaussian elimination or the Lanczos algorithm. On a quantum computer, however, the quantum phase algorithm^38^,^39^,^40^ can project the simplex states 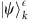 onto the eigenspaces of the Dirac operator *B*^*ε*^ and find corresponding eigenvalues to accuracy *δ* in time 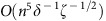, where as above ζ is the accuracy to which we choose to construct the simplex state. The factor of *n*^5^ arises because the quantum phase algorithm applied to an *n*-sparse matrix requires time *n*^3^/*δ*^−1^: the extra factor of *n*^2^ arises because it takes time *O*(*k*^2^) to evaluate the projector 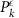 onto the subspace of *k* simplices.

The algorithm also identifies the dimension of the eigenspaces of the Dirac operator and combinatorial Laplacian in time 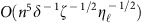, where 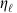 is equal to the dimension 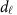 of the 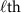 eigenspace divided by 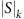, the dimension of the *k*-simplex space. The *k*th Betti number *β*_*k*_ is equal to the dimension of the kernel of Δ_*k*_. The algorithm allows us to construct the full decomposition of the simplicial complex in terms of eigenvectors and eigenvalues of the combinatorial Laplacian, yielding useful geometric information such as harmonic forms. Monitoring how the eigenvalues and eigenspaces of the combinatorial Laplacian change as *ε* changes provides geometric information about how various topological features such as connected components, holes and voids come into existence and disappear as the grouping scale changes^16^,^17^,^44^.

## Discussion

This paper extended methods of quantum machine learning to topological data analysis. Homology is a powerful topological tool. The representatives of the homology classes for different *k* define the connected components of the simplicial complex, holes, voids and so on. The Betti numbers count the number of connected components, holes, voids and so on. Varying the simplicial scale *ε* and tracking how Betti numbers change as function of *ε* reveals how topological features come into existence and go away as the data is analysed at different length scales. Our algorithm also reveals how the structure of the eigenspaces and eigenvalues of the combinatorial Laplacian changes as a function of *ε*. This ‘persistent geometry' reveals features of the data such as rate of change of harmonic forms over different simplicial scales.

The underlying methods of our quantum algorithms are similar to those in other big quantum data algorithms^19^,^20^,^21^. The primary difference between the topological and geometrical algorithms presented here, and algorithms for, for example, constructing clusters^19^, principal components^20^, and support vector machines^21^, is that our topological algorithms require only a small qRAM of size *O*(*n*^2^). Consequently, even when the full qRAM resources are included in the accounting of the computational complexity of the algorithms, the topological algorithms require only an amount of computational resources polynomical in the number of data points, while the best existing classical algorithms for answering the same questions require exponential resources.

To recapitulate the steps of the algorithm: First, the quantum data is processed using standard techniques of quantum computation: distances between points are evaluated, simplices of neighbouring points are identified, and a simplicial complex is constructed. The simplicial complex depends on the grouping scale *ε*. We construct a quantum state that represents the filtration of the complex—the set of simplicial complexes, related by inclusion, for different *ε*. This quantum state contains exponentially fewer qubits than the number of bits required to describe the classical filtration of the complex. Second, we use the quantum phase algorithm^41^,^42^,^43^ to calculate the eigenvalues and to construct the eigenspaces of the combinatorial Laplacian at each scale *ε*. The dimension of the kernel of the combinatorial Laplacian for *k* simplices is the *k*th Betti number. In addition, this construction gives us geometric information about the data set.

Classical algorithms for performing the full persistent homology over a space with *n* points over all scales *k* take time *O*(2^2*n*^): there are 2^*n*^ possible simplices, and evaluating kernels and images of the boundary map via Gaussian elimination for sparse matrices takes time that goes as the square of the dimension of the space of simplices. By contrast, the quantum algorithm for constructing the Betti numbers and for decomposing the simplicial complex in terms of eigenvalues and eigenvectors of the combinatorial Laplacian takes time *O*(*n*^5^), compared with *O*(2^2*n*^) for classical algorithms. The eigenvectors of the kernels of the combinatorial Laplacian are related to the representatives of the *k*th homology class via a boundary term. How to extend the quantum algorithms given here to construct the full barcode of persistent homology and to construct the representatives of the homology class directly is an open question. It would also be interesting to extend the quantum algorithmic methods developed here to further algebraic and combinatorial problems, for example, Morse theory.

## Methods

### Overview

In this section we provide further details of distance evaluation, simplex state construction, and the form of the Dirac operator and the combinatorial Laplacian.

### State preparation and distance evaluation

Topological analysis of the data requires distances between data points. Assume that the data set contains *n* points together with the *n*(*n*−1)/2 distances between them. The data is stored in qRAM or qRAM^35^,^36^,^37^, so that the algorithm can access the data in quantum parallel. The essential feature of a qRAM is that it preserves quantum coherence: the qRAM maps a quantum superposition of inputs 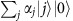 to a quantum superposition of outputs 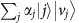. Note that a quantum RAM is potentially significantly easier to construct than a full-blown quantum computer. The storage medium of a quantum RAM can be essentially classical: indeed, a single photon reflected off a compact disk encodes in its quantum state all the bits of information stored in the mirrors on the disk. In addition to a classical storage medium such as a CD, a qRAM contains quantum switches that can be opened in quantum superposition to access that information in quantum parallel. Each call to an *N*-bit qRAM requires log_2_ *N* quantum operations. Quantum RAMS have been designed, and prototypes have been constructed^35^,^36^,^37^. In contrast to other big quantum data algorithms^31^,^32^,^33^, the size of the qRAM required to perform topological and geometric analysis is relatively small: because the computational complexity of classical algorithms for persistent homology scales as *O*(2^2*n*^), while the quantum algorithms require only *O*(*n*^2^) bits worth of qRAM, a significant quantum advantage could be obtained by a qRAM with hundreds to thousands of bits.

As an alternative to being presented with the pre-calculated distances, the data set could consist of *n d*-dimensional vectors 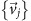 over the complex numbers, and we can use the qRAM to construct the distances 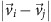 between the *i*th and *j*th vectors^31^. Finally, the distances can be presented as the output of a quantum computation. In all cases, our quantum algorithms for topological and geometric analysis operate by accessing the distances in quantum parallel. Big quantum data analysis works by mapping each vector 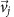 to a quantum state 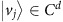, and the entire database to a quantum state 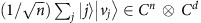. A quantum RAM can be queried in quantum parallel: given an input state 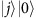, it produces the output state 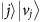, where 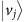 is normalized quantum state proportional to the vector 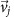. Such a quantum state can be encoded using *O*(log_2_(*nd*)) quantum bits, and 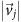 is the norm of the vector.

If we have not been given the *n*(*n*−1)/2 distances directly in qRAM, the next ingredient of the quantum algorithm is the ability to evaluate inner products and distances between vectors. In refs 20, 31, 32, 33 it is shown how the access to vectors in quantum superposition: the ability to create the quantum states corresponding to the vectors translates into the ability to estimate 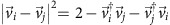. That is, we can construct a quantum circuit that takes as input the state 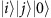 and produces as output the state 
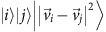
, where the third register contains an estimate of the distance between 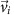 and 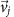. To estimate the distance to accuracy *δ* takes *O*(*δ*^−1^) quantum memory calls and *O*(*δ*^−1^ quantum operations. As with the qRAM, the circuit to evaluate distances operates in quantum parallel.

### Simplex state construction

To elucidate the construction of the *k*-simplex states (1), we look more closely into the implementation of Grover's algorithm to understand when it succeeds in constructing the *k*-simplex state, and how it fails. Start from a superposition 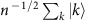 over all values of *k*. Performing simplex construction in parallel via Grover's algorithm with the membership function 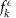 yields the full simplex state at scale *ε*:


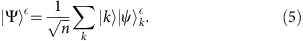


By adding ancillae as above, we can also construct the uniform mixture over all values of *k* and all *k* simplices: 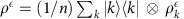. More precisely, if we run the quantum search procedure for a time ζ^−1/2^, we will obtain the state


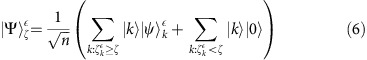


that contains the simplex states 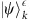 for which 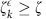 and which returns a null result 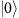 for the simplex states for which 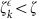. For small *ε*—where only a small fraction of all possible simplices lie within the complex—and fixed ζ, the simplex state 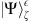 will contain the actual simplex states 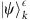 only for small *k*. As *ε* becomes larger and larger, higher and higher *k*-simplex states enter the filtration and 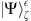 will contain more and more of the *k*-simplex states.

Constructing the simplex state in quantum parallel at *m* different grouping scales *ε*_*i*_ yields the filtration state


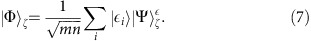


The filtration state 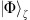 contains the entire filtration of the simplicial complex in quantum superposition. The quantum filtration state contains exponentially fewer quantum bits than the number of classical bits required to describe the classical filtration of the complex: log*m* qubits are required to register the grouping scale *ε*, and *n* qubits are required to label the simplices. 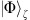 takes time *O*(ζ^−1/2^*n*^2^ log(*m*)) to construct. By contrast, a classical description of the filtration of the simplicial complex requires *O*(2^*n*^) bits.

### Explicit form of the Dirac operator and simplicial Laplacian

Here we present the full matrix form of the Dirac operator *B*^*ε*^ and the combinatorial Laplacian (*B*^*ε*^)^2^. The Dirac operator is


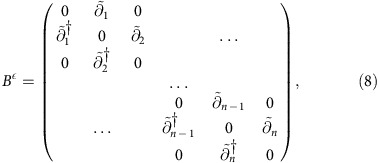


where as above 
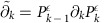
 is the boundary map confined to the simplicial subspace 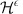. It is straightforward to verify that the Dirac operator is *n*-sparse.

The combinatorial Laplacian is obtained by squaring the Dirac operator:


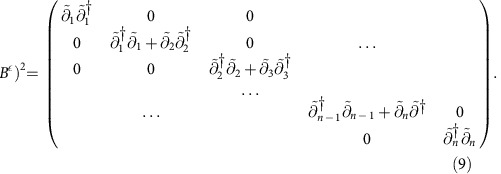


The quantum algorithm operates by diagonalizing the Dirac operator.

## Additional information

**How to cite this article:** Lloyd, S. *et al*. Quantum algorithms for topological and geometric analysis of data. *Nat. Commun.* 7:10138 doi: 10.1038/ncomms10138 (2016).

## Figures and Tables

**Table 1:** Computational cost comparison.

**Procedural steps**	**Classical cost**	**Quantum cost**
Input pairwise distances, *n* points	*O*(*n*^2^) bits	*O*(*n*^2^) bits
Construct simplicial complex	*O*(2^*n*^) ops	*O*(*n*^2^) ops on *O*(*n*) qubits
Diagonalize Laplacian/find Betti numbers	*O*(2^2*n*^ log(1/*δ*)) ops	*O*(*n*^5^/*δ*) quantum ops

*δ* is the multiplicative accuracy to which the Betti numbers and the eigenvalues of the combinatorial Laplacian are determined. Note the trade-off between the exponential quantum speed-up and accuracy: the quantum algorithms obtain an exponential speed-up over classical algorithms but provide an accuracy that scales polynomially in 1/*δ* rather than exponentially. This feature arises from the nature of the quantum phase estimation/matrix inversion algorithms, which obtain their exponential speed-up by estimating eigenvectors and eigenvalues using a ‘pointer-variable' measurement interaction^38^,^39^,^40^. By contrast, classical algorithms need only keep *O*(log(1/*δ*)) bits of precision, but must perform *O*(2^2*n*^) steps to diagonalize 2^*n*^ × 2^*n*^ sparse matrices.
